# Arachidonic Acid Is a Safe and Efficacious Schistosomicide, and an Endoschistosomicide in Natural and Experimental Infections, and Cysteine Peptidase Vaccinated Hosts

**DOI:** 10.3389/fimmu.2020.609994

**Published:** 2020-11-17

**Authors:** Hatem Tallima, Violette S. Hanna, Rashika El Ridi

**Affiliations:** ^1^Zoology Department, Faculty of Science, Cairo University, Giza, Egypt; ^2^Department of Chemistry, School of Science and Engineering, American University in Cairo, New Cairo, Cairo, Egypt

**Keywords:** *Schistosoma mansoni*, *Schistosoma haematobium*, arachidonic acid, schistosomicide, endoschistosomicide, cysteine peptidase vaccine

## Abstract

Blood flukes of the genus Schistosoma are covered by a protective heptalaminated, double lipid bilayer surface membrane. Large amounts of sphingomyelin (SM) in the outer leaflet form with surrounding water molecules a tight hydrogen bond barrier, which allows entry of nutrients and prevents access of host immune effectors. Excessive hydrolysis of SM to phosphoryl choline and ceramide *via* activation of the parasite tegument-associated neutral sphingomyelinase (nSMase) with the polyunsaturated fatty acid, arachidonic acid (ARA) leads to parasite death, *via* allowing exposure of apical membrane antigens to antibody-dependent cell-mediated cytotoxicity (ADCC), and accumulation of the pro-apoptotic ceramide. Surface membrane nSMase represents, thus, a worm Achilles heel, and ARA a valid schistosomicide. Several experiments conducted *in vitro* using larval, juvenile, and adult *Schistosoma mansoni* and *Schistosoma haematobium* documented ARA schistosomicidal potential. Arachidonic acid schistosomicidal action was shown to be safe and efficacious in mice and hamsters infected with *S. mansoni* and *S. haematobium*, respectively, and in children with light *S. mansoni* infection. A combination of praziquantel and ARA led to outstanding cure rates in children with heavy *S. mansoni* infection. Additionally, ample evidence was obtained for the powerful ARA ovocidal potential *in vivo* and *in vitro* against *S. mansoni* and *S. haematobium* liver and intestine eggs. Studies documented ARA as an endogenous schistosomicide in the final mammalian and intermediate snail hosts, and in mice and hamsters, immunized with the cysteine peptidase-based vaccine. These findings together support our advocating the nutrient ARA as the safe and efficacious schistosomicide of the future.

## Introduction

The intrammalian stage of schistosomes that infect humans, notably *Schistosoma mansoni* and *Schistosoma haematobium* displays an unusual heptalaminated, double lipid layer surface membrane, an evident adaptation to intravascular life (1, 2). Membranes were isolated from the tegument of adult *S. mansoni* by spontaneous release into phosphate-buffered saline, with or without vortexing, and by removal from the parasite’s surface using poly-lysine beads. The phospholipids showed a typical plasma membrane-like profile, except for high (approximately 20%) sphingomyelin (SM) content (3). Fluorescent microscopy and fluorescent recovery after photobleaching techniques indicated that SM localizes to the outer monolayer, and sphingosine and ceramide within or below the outer membrane (4), and demonstrated presence of a SM cycle in *S. mansoni* adult males (5). Assays carried out for sphingomyelinase activity were unable to detect SM breakdown at acidic pH, but did detect activity at pH 7.4. This activity was stimulated by arachidonic acid (ARA) and MgCl_2_ (5).

**Figure 1:**
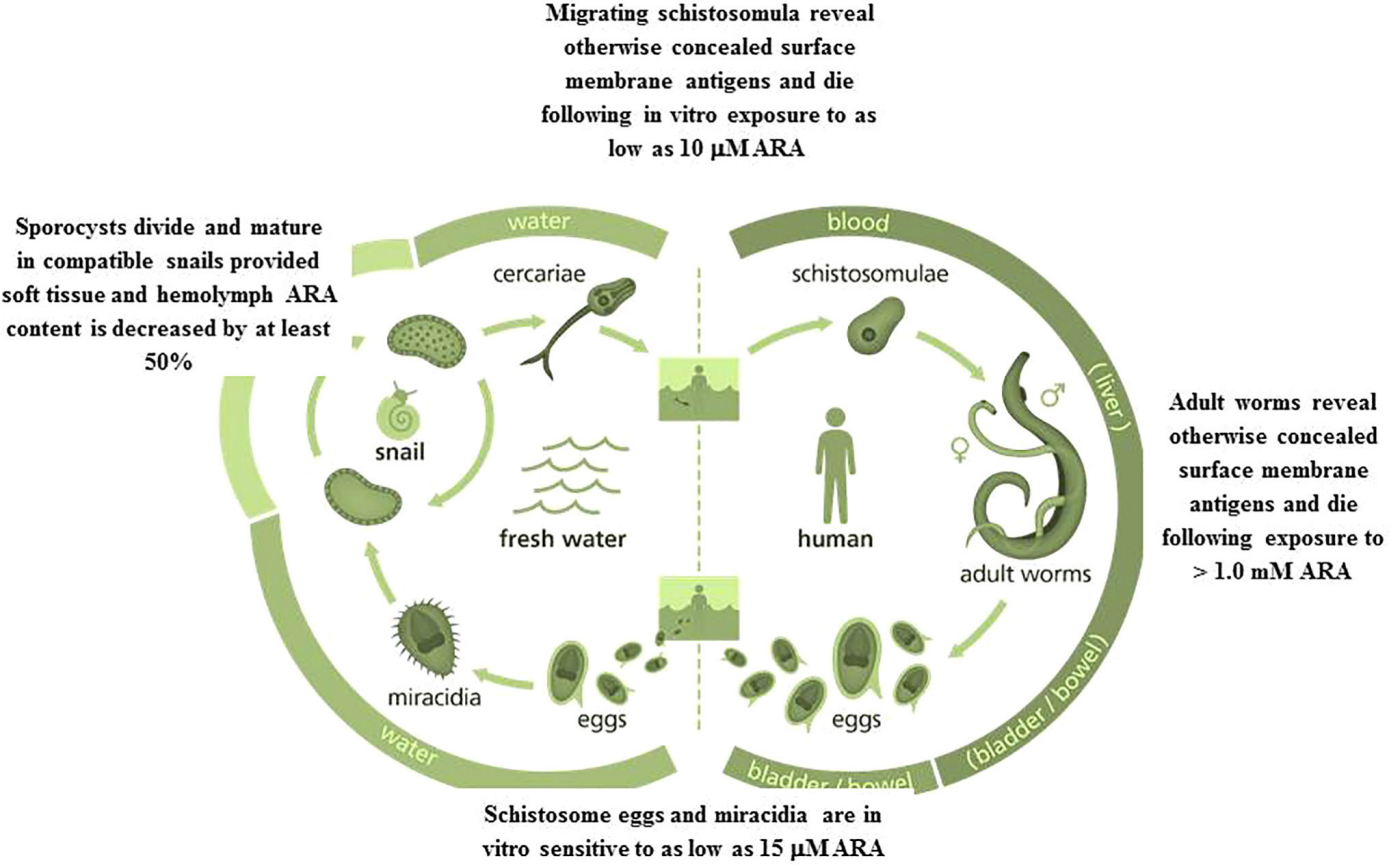
Eggs, miracidia, sporocysts, migrating schistosomula, and adult worms are all sensitive to ARA exposure *in vitro* and *in vivo*. Image credit: Genome Research Limited.

Lung stage, and adult *S. mansoni* more than *S*. *haematobium* surface membrane outer leaflet is also rich in cholesterol, which can be totally extracted following incubation for 2 h in the presence of 40 mM methyl-β-cyclodextrin (MBCD) (6–8). Apical lipid bilayer total cholesterol extraction, as judged by filipin staining assay, led to exposure of surface membrane antigens to antibody binding in 70 and 50% of adult *S*. *mansoni* and *S. haematobium*, respectively (8), and *ex vivo* lung-stage larvae of *S. mansoni* but not *S*. *haematobium* (9, 10). These findings suggested that cholesterol is an essential, yet not the sole, factor in sequestration of schistosomes surface membrane antigens (6–10). Incubation of *S. mansoni* and *S. haematobium* lung-stage larvae in the presence of mono (olive oil) or poly (PUFAs) unsaturated fatty acids was shown to elicit SM hydrolysis, and exposure of the worm otherwise concealed antigens to antibody binding in the indirect membrane immunofluorescence (IF) test, independently of cholesterol extraction (9, 10). In 2006, we predicted and provided evidence for the existence of a schistosome tegument-associated, Mg^++^-dependent, neutral sphingomyelinase (nSMase) (10), which was later sequenced and identified by Berriman et al. in 2009, with subsequent improvements by Protasio et al. (11, 12). In 2011, the partial cloning and sequencing, enzymatic activity, and immunolocalization of *S*. *haematobium* nSMase were reported (13, 14). Blasting of our results with *S. mansoni* nSMase sequence revealed 97% identity (14). The genome sequence of full-length *S*. *haematobium* nSMase was published (15, 16), fully confirming our results. Incubation of *S. mansoni* and *S. haematobium ex vivo* lung-stage larvae in the presence of nSMase inhibitors and stimulators indicated that nSMase activity leads to hydrolysis of some SM molecules allowing entry of nutrients but not host antibodies or immune effectors (9, 10, 17). The PUFA, ARA, is a prominent nSMase activator (18–20). Lung-stage and adult *S. mansoni* and *S. haematobium* excessive nSMase stimulation by ARA led to worm surface membrane exposure to antibody binding in IF assays, and eventual death (9, 10, 21). Parasite tegument-associated nSMase represents, thus, a worm genuine Achilles heel, and ARA a promising schistosomicide. The present article documents ARA schistosomicidal safety and efficacy (**Table 1**), and endogenous antischistosomal potential against the different parasite stages in the final and intermediate hosts (**Figure 1**).

**Table 1:** *In vivo* arachidonic acid therapeutic action.

Experimental host	Schistosome species			Ref
	*Schistosoma mansoni* ^a^	*Schistosoma haematobium* ^b^		
**Mice**				**(**21**)**
ARA dose	1,000 mg/kg	1,000 mg/kg		
Worm burden decrease	*P* = 0.007; 37.9%	*P* = 0.003; 57.7%		
ARA dose	300 mg/kg/d ^c^ for 15 ^d^	300 mg/kg/d for 15 d		
Worm burden decrease	*P* = 0.001;63.6%	*P* = 0.007; 81.4%		
**Hamsters**				**(**22**)**
ARA dose	300 + 2,500 mg/kg/d over 2 d	300 + 2,500 mg/kg/d over 2 d		
Worm burden decrease	*P* = 0.017; 78.9%	*P* = 0.0002; 50.6%		
**Children**^c^	**Number of cured/total number of treated children (cure rate %)**			
	**Light infection**	**Moderate infection**	**Heavy infection**	**Ref**
**Low endemicity region**				**(**23**)**
PZQ 40 mg/kg once	12/14 (85)	5/6 (83)		
ARA 10 mg/kg/d for 15 d	11/14 (78)	4/9 (44)		
PZQ + ARA	14/16 (87)	7/7 (100)		
**High endemicity region**				**(**24**)**
PZQ 40 mg/kg once	19/32 (60)	11/26 (42)	3/15 (20)	
ARA 10 mg/kg/d for 15 d	12/24 (50)	3/23 (13)	3/14 (21%)	
PZQ + ARA	19/23 (83)	13/23 (57)	11/14 (78)	

a Mice and hamsters infected with S. mansoni were ARA-treated 6 weeks after infection.

b Mice and hamsters infected with S. haematobium were ARA-treated 12 weeks after infection.

c Children were infected with S. mansoni.

d d = day.

## Arachidonic Acid Schistosomicidal Activity *In Vitro* and in Experimental Hosts

Incubation of *ex vivo S. mansoni* and *S. haematobium* juvenile and adult stages in the presence of pure ARA was investigated in terms of ARA concentration, fetal calf serum (FCS) percentage in the culture medium, and exposure time (21). Hundred percent of 21- day-old *S. mansoni* and *S. haematobium* were killed after 2, and adults after 5 h exposure to 2.5 mM ARA/20% FCS, indicating the higher susceptibility of juvenile stages. Irreversible death of adult worms cultured in 100% FCS required 5 h incubation in the presence of 10 mM ARA, likely because of ARA binding to serum albumin, allowing only few millimolar concentrations to be stabilized in an aqueous environment (25). Propensity of ARA to bind to serum albumin explains why adult worms strive in human blood, where unesterified free ARA is in the normal range of less than 1 mM 300 microg/mL (25–27), and the difficulty of increasing serum ARA concentrations to the coveted schistosomicidal levels (21). No worm death was observed when assays were performed in the presence of nSMase specific inhibitors, suggesting SM hydrolysis is a major ARA-mediated schistosomicidal mechanism, resulting into tegument disintegration and destruction as judged by scanning and transmission electron microscopy (21). The ARA-mediated effects were more pronounced in juvenile than adult worms and *S. haematobium* than *S. mansoni* (21). Lung stage larvae were killed after exposure to 10-20 µM ARA (9, 10, 21). The data indicate that parasites at the onset of host infection are susceptible to ARA direct schistosomicidal action provided a relatively high level of surrounding free ARA (**Figure 1**). Unesterified ARA concentration reaches 3 to 6 µM in healthy and >50 µM in inflamed skin, and up to 500 µM in serum under certain pathophysiological conditions (25, 28). Arachidonic acid physiological concentrations at sites of inflammation, such as granulomatous liver, may reach 100 µM (25, 29), overlapping with the concentration required to *in vitro* kill the great majority of *S. mansoni* eggs obtained from 7 week-infected hamster liver and small intestine (30). Accordingly, differential susceptibility and resistance to initial cercarial infection, and variations in number of excreted schistosome eggs in children and adults might well be attributed to available free ARA levels in skin, serum, liver, and intestine.

In accord with the difficulty of raising serum ARA content to schistosomicidal levels, a single ARA oral dose calculated to elevate the plasma level of schistosome infected BALB/c and C57BL/6 mice to approximately 12 mM (500 mg/kg, targeting the lung stage), or 30 mM (1,000 mg/kg, targeting the adult stage) led to significant (*P* < 0.05 to < 0.01), yet modest reduction of 39.3 and 37.9% in *S. mansoni* and 57% in *S. haematobium* total, and male and female worm burden. However, mouse ingestion of 300 mg/kg ARA in milk for 15 days led to highly significant (*P* < 0.001) reduction of approximately 60.0% in *S. mansoni* and 81.4% in *S. haematobium* total worm burden (21). In hamsters as well, oral ARA administration after patency of *S*. *mansoni* and *S. haematobium* led to dose-dependent (300, 1200, or 2500 mg/kg, over one or two days) highly significant (*P* < 0.02 to <0.001) reduction in worm burden accompanied by a significant (*P* ******< 0.05) decrease in worm egg load (**Table 1**). ARA-mediated protection against hamster schistosomiasis was associated with high titers of serum antibodies to tegumental antigens, suggesting a different ARA schistosomicidal mechanism besides, or independently, of direct parasiticidal activity. Serum ARA levels higher than 1 mM readily activates parasite tegument-associated nSMase, leading to considerable SM hydrolysis, and exposure of surface membrane antigens. Binding of host antibodies followed by antibody-dependent cell-mediated cytotoxicity (ADCC) may compete with, and perhaps overcome the worm repair mechanisms. In support, serum antibodies from patently infected and ARA-treated hamsters readily bound to the surface membrane of ARA-exposed adult worms, as judged by indirect membrane immunofluorescence. More importantly, incubation of healthy adult worms in the presence of anti-parasite tegument antibodies and peripheral blood mononuclear cells significantly enhanced ARA-mediated adult parasite demise *in vitro* (22). The data together showed that ARA schistosomicidal effect in laboratory animals is mediated and/or enhanced by immune effectors, and is entirely safe (22). Indeed, in every experimental setting, no adverse effects were associated with long term ARA-administered mice (21) or rats (31) regarding appetite, posture, activity, survival, or gross pathology.

## Arachidonic Acid Schistosomicidal Activity in Children Infected With *S. mansoni*

The efficacy and safety of ARA in treatment of school-age children infected with *S. mansoni* were examined in *S. mansoni* low (23) and high (24) endemicity regions. Children were given a single oral dose of praziquantel (PZQ) (40 mg/kg) on the first day of treatment and placebo (Corn-Soy, and 1 g VegCap) provided by DSM (Heerlen, Netherlands) for the next 3 weeks (5 doses/week); ARA (10 mg/kg per day) using ARASCO oral capsules containing 1 g VegCap and 396 mg ARA (DSM), for 15 days over 3 weeks (5 days/week); or PZQ (40 mg/kg) on the first day of treatment and 15 doses of ARA (10 mg/kg per day for 5 doses/week). No single child reported any adverse reactions during or after treatment with ARA, contrary to a substantial majority of children treated with PZQ reported transient headache, dizziness, abdominal pain, nausea, and diarrhea. Symptoms disappeared the next day, and children received placebo or ARA capsule without any additional malaise. The studies showed that long term ARA administration in humans is entirely safe, as was previously recorded in experimental hosts (21, 31), and despite failure in eliciting significant changes in circulating PUFAs parameters, its therapeutic efficacy in *S. mansoni*-infected children was highly comparable to PZQ (23, 24). Indeed, ARA was therapeutically comparable to PZQ regarding treatment efficacy of light and heavy infection (**Table 1**).

Curiously, therapeutic efficacy of ARA as well as PZQ considerably varied among children in *S. mansoni* low and high endemicity regions, Menoufiya and Kafr El Sheikh, respectively (**Table 1**) (23, 24, 32). The results concurred with numerous research articles reporting association between PZQ efficacy with baseline infection intensity and levels of schistosomiasis prevalence and endemicity (33, 34).

Low schistosomiasis cure rates in heavy infection in areas of high endemicity are not due to resistance to PZQ per se, since they were recorded following monotherapy with PZQ and ARA. Instead we have proposed that continuous PZQ use during intensive and repeated mass treatment campaigns leads to selection of worms with tighter outer lipid bilayer armor, due to SM accumulation attributed to high SM synthesis, or decreased degradation consequent to reduced parasite tegument-associated nSMase content or activity. Circumstantial ample evidence was recorded whereby combination of the nSMase activator ARA and PZQ led to 100% cure rates of light and moderate infections in low *S. mansoni* endemicity regions, and highly significant (*P* < 0.0001) increase in cure rates of low, moderate, and heavy infections in children resident in Kafr El Sheikh (**Table 1**) (23, 24, 32). The findings have prompted DSM to sponsor a patent advocating combination of ARA and PZQ for the prevention and treatment of schistosomiasis mansoni (35). Trials are needed to demonstrate the expected higher ARA therapeutic activity in treatment of schistosomiasis haematobium in children and adults. ARA intake for schistosomiasis therapy is entirely safe. In humans, cell membrane ARA may undergo oxidation by different enzymes leading to a plethora of pro-inflammatory and anti-inflammatory resolving mediators (25, 36). ARA obtained from food or nutraceutical capsules does not undergo oxidation. It is incorporated in phospholipids in the cells’ cytosol, or remains in the serum bound to albumin or in an unesterified free form (25, 36). Moreover, a systematic review reported that increased ARA intake in adults up to 1,000–1,500 mg/day had no adverse effects, failed to increase the concentrations of many inflammatory markers, and was linked to reduced inflammation (37, 38).

## Arachidonic Acid Is an Endoschistosomicide in the Final Mammalian Hosts

The pioneer study of Amaral et al. (39) reported that the water rat, *Nectomys squamipes*, a wild reservoir of *S. mansoni* in Brazil is continuously subjected to natural infection, yet displays low worm and worm egg burdens and limited liver histopathological changes, associated with accumulation of PUFAs, namely ARA, in numerous lipid droplets in the liver. This association may lead to speculate that high ARA levels impair the development of a substantial percentage of invading parasites and, most importantly, the viability of liver-trapped eggs. The direct ARA *in vitro* schistosomicidal action on lung-stage larvae, juvenile and adult schistosomes was amply documented, provided the worms are exposed to a high ARA concentration (9, 10, 21), which was apparently available in the water-rat (39). We have recently showed that exposure of viable eggs obtained from the liver and small intestine of *S. mansoni* infected mice to 15–500 µM ARA concentrations induced a significant (*P* < 0.005) percentage of eggs to hatch before exposure to water and strong light and release miracidia, which all were found dead. Of note, exposure to ARA concentrations as low as 15 µM elicited significant (*P* < 0.05) increase in the percentage of eggs that failed to hatch after exposure to water and light (30) (**Figure 1**). These findings indicate that ARA is ovocidal and larvacidal, and give support to the direct correlation between low parasite egg burden and limited liver histopathological changes with liver ARA depots in the water rat, *N. squamipes* (39, 40).

Differently from the water-rat and laboratory mice, ARA organ and serum concentrations are high in uninfected laboratory rats, *Rattus norvegicus*. Infection with up to 200 cercariae of *S. mansoni* was met with continuous exposure to up to 1 µM serum free, unesterified ARA, lethal enough to 2–5 day old migrating larvae, associated with complete inhibition of parasite development, maturation, and egg deposition (41). Infection of hamsters and CD-1mice with 200 cercariae of *S. haematobium* was met with continuous exposure to up to 1.0 µM serum free, unesterified ARA in hamsters and lower concentrations in mice. Yet, ARA serum levels significantly (*P* < 0.05) declined and increased in hamsters and mice, respectively during week 1 and 2 post infection, perhaps explaining the susceptibility of hamsters and semi-permissiveness of mice to S. *haematobium* (41). Serum concentrations in the range of 1.0 µM might not be sufficient to directly kill the developing worm, but readily leads to tegument-associated nSMase activation and apical SM hydrolysis, with subsequent parasite disintegration due to exposure of otherwise concealed surface membrane antigens to antibodies and antibody-mediated cell cytotoxicity, and/or release of intra worm apoptotic signals (22, 29, 41). Like the water- and laboratory rat, severe combined immunodeficient (SCID) mice are enriched in serum ARA. *Schistosoma japonicum* larvae invading SCID mice are met with serum ARA concentration three folds higher than in immunocompetent BALB/c mice, perhaps explaining the poor parasite recovery, maturation and egg production consistently recorded in immunodeficient mice (42).

Curiously, the anti-schistosomal activities of compound 12, a lipophilic derivative of Sclareol, a plant-derived diterpenoid widely used as a fragrance and flavoring substance, were reported to be specifically associated with higher abundance of ARA within the metabolite pool of treated worms when compared to controls (43), additionally implicating ARA as a parasite endogenous schistosomicide. On another aspect, exosome-like vesicles derived from adult *S. mansoni* were found to be internalized by vascular endothelial cells and monocytes and to powerfully up regulate ARA metabolism (44), a remarkable host defense mechanism as ARA and other unsaturated fatty acids and some of their metabolites function as endogenous antimicrobial molecules (45).

## Arachidonic Acid Is Endoschistosomicidal in the Intermediate Snail Hosts

Laboratory bred *Biomphalaria alexandrina* and *Bulinus truncatus* snails were exposed to miracidia of laboratory-maintained *S. mansoni* and *S. haematobium* respectively. Snails were examined for presence or lack of infection association with soft tissue and hemolymph content of palmitic, oleic, linoleic, and arachidonic acid, assayed by ultra-performance liquid chromatography-tandem mass spectrometry (UPLC-MS/MS). Soft tissue palmitic, oleic, linoleic and arachidonic acid content reached a mean of, respectively 400, 1,000, 40, and 1,000 ng/mg protein of *B. alexandrina* and 150, 100, 40, and 400 ng/mg protein of *B*. *truncatus* naïve snails. Hemolymph palmitic acid content was 2,000 and 1,500 ng/mg protein of *B. alexandrina* and *B*. *truncatus*, respectively. Hemolymph oleic, linoleic and arachidonic acid content reached a mean of around 300 ng/mg protein of *B. alexandrina* and 150 ng/mg protein of *B*. *truncatus* naïve snails. Schistosome infection led to no change or some increase in palmitic acid content in snail soft tissues and hemolymph. Conversely, infection failed to proceed in any snail, algae or lettuce-fed, except when associated with highly significant (up to *P* < 0.0001) decrease of 50 to 60% in soft tissue and hemolymph content of assayed mono and polyunsaturated fatty acids (46). Of note, invading schistosomes were indifferent to the levels of palmitic acid in snail soft tissue and hemolymph. It is because PUFAs, and not saturated fatty acids, that function as endogenous antimicrobial molecules (45). The results together suggest *S. haematobium*, known for their higher sensitivity to PUFAs (9, 10, 21, 41), would have never approached *B. truncatus* if it carried the PUFAs levels of *B. alexandrina*. Second, manipulating the schistosome intermediate snail hosts diet towards excessive accumulation of PUFAs in soft tissue and hemolymph would interfere with intra snail schistosome development and transmission of infection. Finally, PUFAs, especially ARA, affect the development of schistosomes in both the intermediate snail and final mammalian hosts (**Figure 1**).

## Arachidonic Acid Is a Critical Mediator of Protection in the Cysteine Peptidase-Based Schistosomiasis Vaccine Model

Immunization of outbred mice or hamsters with schistosome molecules that are both excretory-secretory products (ESP) and type 2 immune responses-inducing, namely cysteine peptidases, consistently and reproducibly elicited highly significant (*P*<0.0001) reduction of 50–60% challenge schistosome worm burden and worm egg load in liver and intestine when compared to unimmunized hosts (30, 47–54). Reduction in worm burden was not observed at the lung-stage, and likely occurs at the liver stage concomitantly with increase in anti-cysteine peptidase antibody titer, and levels of ARA in serum and liver (30). Serum ARA levels were significantly (*P* < 0.05) higher than infection controls in the cysteine peptidase-immunized mouse groups on day 17 post infection. Levels of ARA in lung and liver of cysteine peptidase -immunized mice were considerably higher as compared to naïve and unimmunized infected control mice in lung at 10 and liver at 17 and 24 days post infection (30). We proposed that immune cells-activating antibodies to the cysteine peptidase immunogens and ARA, together, mediate demise of juvenile worms. The importance of antibodies in mediating the cysteine peptidase vaccine resistance to infection was demonstrated in the *S. mansoni* (30) and the *Ancylostoma ceylanicum* (55) models. It has been reported that free ARA is readily incorporated by *S. mansoni* (56), and likely activates the parasite voltage-gated channels, and enzymes, namely tegument-associated nSMase with subsequent apical SM hydrolysis, and exposure of the otherwise hidden surface membrane antigens to antibody- mediated effector functions, and parasite demise. Sphingomyelin degradation leads to accumulation of intra worm apoptotic signals (57). Arachidonic acid, a powerful host phagocyte NADPH oxidase activator, contributes to inflammation *via* eliciting massive production of reactive oxygen (ROS) and nitrogen (RNS) species (58), which are harmful to the schistosomes and perhaps responsible for egg attrition, immediately upon trapping. Furthermore, ARA peroxidation following exposure to ROS or RNS generates isoprostanes, responsible of oxidative stress and injury (36, 45). Arachidonic acid mediated oxidative stress is however efficiently counteracted by host and schistosome anti-oxidants (59, 60), thus explaining the protection ceiling of the cysteine peptidase-based schistosomiasis vaccine (30, 47–54.

## Conclusions

The reported findings suggest that ARA should be considered for safe and efficacious therapy of human schistosomiasis, besides being a critical nutrient. Accordingly, every effort should be made to encourage ARA production at the commercial level in developing countries as the demands of Middle East and African markets are expected to increasingly augment.

## Author Contributions

All authors drafted, revised, edited, and approved the final version of the manuscript.

## Conflict of Interest

The authors declare that the research was conducted in the absence of any commercial or financial relationships that could be construed as a potential conflict of interest.
